# Neurobrucellosis presenting with concurrent paraplegia and global aphasia in an elderly butcher: A case report and literature review

**DOI:** 10.1016/j.idcr.2026.e02520

**Published:** 2026-02-09

**Authors:** Davood Sanaei Delir Zavaragh, Negar Abbasi Jamaat, Yasamin Pourabedini Aghdam, Amir Ehtemami, Kamyar Khazaei

**Affiliations:** a Department of Infectious Diseases, Farhikhtegan Hospital, TeMS.C., Islamic Azad University, Tehran, Iran; b Iran Azad University of Medical Sciences, Tehran, Iran

**Keywords:** Brucellosis, Neurobrucellosis, Brucella melitensis, Paraplegia, Aphasia, Cerebrospinal fluid

## Abstract

**Background:**

Brucellosis is a prevalent zoonotic infection and presents with wide range of clinical manifestations, including rare but severe neurological complications.

**Case presentation:**

We report the case of a 68-year-old male butcher presenting with global aphasia, paraplegia, incontinence, excessive sweating two weeks prior to admission, and significant weight loss over eight months. Initial evaluations, including brain CT, lumbar puncture, and neurological studies were inconclusive. Spinal MRI showed L1-L2 spondylodiscitis. Serological tests, cerebrospinal fluid culture, and positive Brucella melitensis PCR supported a neurobrucellosis diagnosis. Treatment with a combination of gentamicin, ceftriaxone, rifampicin, and doxycycline was initiated, followed by outpatient therapy with rifampicin, doxycycline, and sulfamethoxazole-trimethoprim for three months. Significant symptom improvement and declining serological titers were observed over two years of follow-up.

**Conclusion:**

This case underscores the diagnostic challenges of neurobrucellosis due to its nonspecific presentation and emphasize on the importance of considering such etiologies in patients with unexplained neurological symptoms, particularly in endemic regions like Iran.

## Highlights

• Neurobrucellosis mimics a wide spectrum from Guillain-Barré-like syndrome to acute cerebrovascular event due to vasculitis.
• Neurobrucellosis merits consideration as differential diagnosis of unexplained neurological manifestations in endemic regions.
• Comprehensive evaluation and awareness of atypical patterns are crucial to avoid misdiagnosis.

## Background

Brucellosis is a zoonotic infection caused by Brucella species. Common initial complaints include fever, arthralgia, weakness, low back pain, gastrointestinal symptoms, and sweating. Inflammatory infiltration in response to Brucella infection can affect any organ, resulting in numerous complications such as neurobrucellesis, which is among the most morbid forms [1]. Neurobrucellosis may clinically present with diverse neurological syndromes, including meningitis, myelopathy, cranial and peripheral neuropathies, or neuropsychiatric changes. Clinical signs may include bilateral neurosensory hearing loss, motor aphasia, hemiparesis, quadriparesis, and confusion [2]. Definitive diagnosis is often quite complicated as there are no pathognomonic clinical features. It relies on a combination of persistent unexplained chronic neurological symptoms, positive serology in blood or cerebrospinal fluid (CSF), and abnormal CSF findings such as elevated protein (>45 mg/dL), altered cellularity (lymphocytosis), and low glucose (CSF/blood glucose ratio <0.4). Isolation of Brucella in culture, when possible, solidly confirms the diagnosis [3].

Treatment requires antibiotics that surpass the blood brain barrier. A triple-drug regimen is generally recommended, as monotherapy or dual therapy may be insufficient. Doxycycline, rifampicin, sulfamethoxazole-trimethoprim, and third generation cephalosporins (with ceftriaxone often used empirically) are capable options, especially in meningoencephalitic forms of neurobrucellosis [3].

We hereby describe an intriguing case of neurobrucellosis with an unusual presentation of aphasia and paraplegia, in which the complex diagnosis was delayed. Our aim was to emphasize on considering neurobrocellosis in patients with unexplained neurological syndromes by addressing this gap in high-risk endemic population.

## Case presentation

A 68-year-old male butcher was referred to Farhikhtegan Hospital with sudden global aphasia, paraplegia, incontinency, and excessive sweating two weeks prior to admission, while he was bedridden. On arrival, decreased level of consciousness was evident as he was lethargic with spontaneous eye opening, but speech was dysarthric, and he was confused and agitated. No lateralizing or focal neurological signs were observed. The patient was in good health until eight months earlier, when he developed back pain in the mid-lumbar region without prior episodes. He suffered from bilateral burning sensation in the hips three months prior to admission followed by progressive lower limb weakness and paraparesis, which advanced to paraplegia over two weeks. His wife reported prominent weight loss of 20 kg during the preceding five months, accompanied by diaphoresis, but denied fevers or chills. The patient had been hospitalized twice (an overall of 3 months) with back pain, fatigue, and weakness of the lower limbs during this course. Several initial evaluations were unrevealing (Table 1). He had no significant past medical history and took no regular medications, although there was a history of drug addiction to an unknown substance.

**Table 1:** Laboratory findings.

	**Result**	**Reference value**
**Hemoglobin**	13.3	
**W.B.C**	6900	
**Neutrophils**	66	
**Lymphocytes**	29	
**ESR 1 h**	67	
**CRP**	90	
**Procalcitonin**	0.11	< 0.5
**Blood Culture**	Negative	
**Urine Analysis**	Normal	
**Urine Culture**	Negative	
**HIV Ab**	Negative	
**PPD**	1	
**IGRA, Quantiferone** **Wright Agglutination**	0.34 1/640	Negative: < 0.35 < 1/80
**Coombs-Wright**	1/1280	< 1/160 in non-endemic regions < 1/320 in endemic regions
**2ME**	1/640	< 1/40

**Fig. 1:**
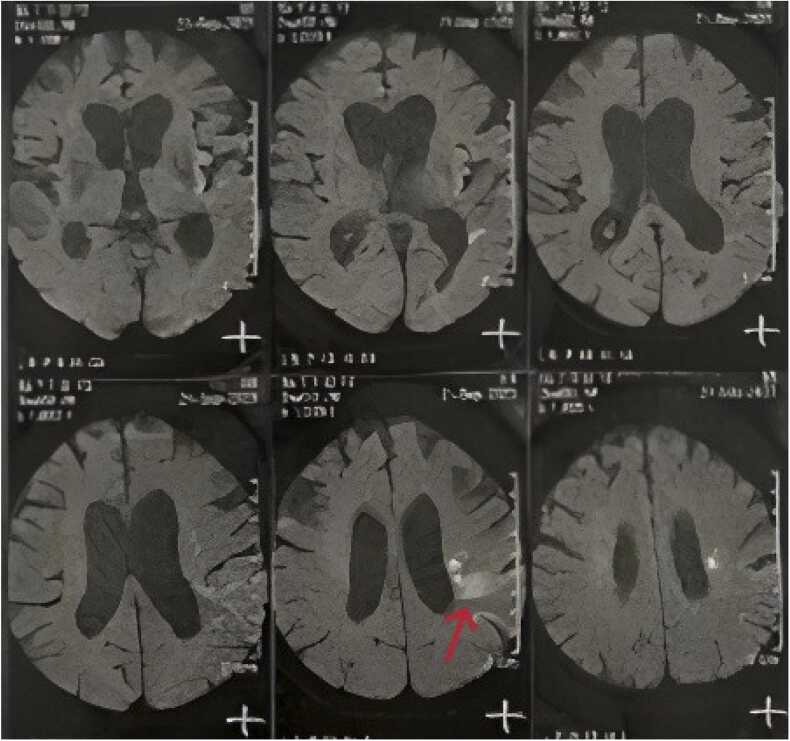
Brain MRI: periventricular, left side centrum semi oval white matter and post central gyrus lesions mostly in favor of microvascular occlusive ischemia in the MCA territory (e.g., lenticulostriate artery) - mild cortical atrophy and ventriculomegaly are also noted.

**Fig. 2:**
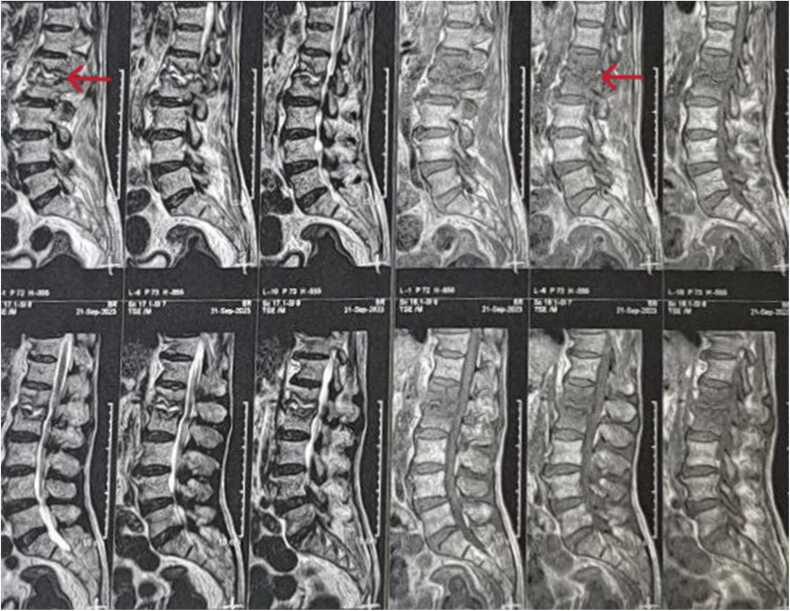
Lumbosacral MRI: disc height loss with sub endplate marrow edema and erosion at L1-L2 level mostly in favor of spondylodiscitis.

Given the paraplegia and dysarthria, neurological causes were considered first. A non-contrast brain CT scan was performed immediately, though no signs of acute cerebrovascular events or tumors were detected. Further diagnostic studies followed. Although lumbar puncture (LP) was performed (Table 2) due to persistent neurological deficits, it did not initially indicate typical features of meningitis. Doppler sonography of the neck arteries showed no significant carotid stenosis and lower extremity Doppler sonography was also was unremarkable. Deep tendon reflex examinations were normal. Electromyography and nerve conduction velocity (EMG-NCV) studies to evaluate polyneuropathies (namely Guillain-Barré syndrome) was inconclusive. ECG, echocardiography, and abdominopelvic sonography were of no significance. Lumbosacral, thoracic, cervical, and brain MRIs were performed due to lack of any progress in the patient's state. In brain MRI, there was apparent microvascular occlusion ischemia in the initial segments of the left middle cerebral artery (MCA) territory (Fig. 1). In addition, Spinal MRI reported multiple anomalies. The most prominent involvement was disc height loss with soft endplate marrow edema, and erosion at T6-T7 and L1-L2 levels (Fig. 2) mostly in favor of spondylodiscitis.

**Table 2:** CSF analysis.

	**Results**	**Reference value**
**Color**	clear	
**Appearance**	colorless	
**Total WBC Count**	< 5	Adult: up to 5 cells
**RBC Count**	30	
**CSF Glucose**	33	50–80
**CSF Protein**	45	15–60
**CSF Culture**	Brucella spp. isolated	
**Wright**	1/40	< 1/80
**Brucella PCR**	positive for Brucella melitensis

Upon repeated CSF analyses, the total white blood cell count remained within normal limits, yet the glucose level was consistently low. This unexpected finding raised our suspicion of an atypical infectious etiology affecting the spinal cord despite the absence of typical meningitis features in attention to the occupational history. After abundant medical work-up and excluding neurological etiologies, serologic tests were requested with a special attention to tuberculosis and brucellosis involving the vertebral column (Table 1). Wright’s titer was 1/640, Coombs Wright was 1/1280, and 2-mercaptoethanol (2ME) was 1/640, consistent with brucellosis. However, blood cultures and PCR for Brucella melitensis were negative. Over time, CSF culture yielded Brucella spp., and PCR confirmed Brucella melitensis (Table 2). Initially, we suspected that Brucella-induced spondylodiscitis at L1-L2 was the primary source that had secondarily progressed to neurobrucellosis through local extension. Thereafter, a vertebral tissue biopsy was obtained but was negative for brucellosis. These findings suggest that the neurological involvement may have resulted from direct hematogenous dissemination to the central nervous system rather than contiguous spread from the spine. Overall, diagnosis of neurobrucellosis was established.

Treatment was initiated with a of combination antibiotic regimen consisting of gentamicin 240 mg/day, ceftriaxone 4 g/day, rifampicin 600 mg/day, doxycycline 200 mg/day for 7 days during hospitalization. Thereafter, outpatient treatment with rifampicin, doxycycline, and sulfamethoxazole-trimethoprim 1600/320 mg/day was extended for 3 months. No adverse effects were reported during antibiotic therapy.

We followed the patient two weeks after discharge, and then in routine 3 month interval via clinical visits and laboratory assessment. He consistently expressed satisfaction with significant amelioration of symptoms relapse or recurrence, accompanied by decreasing trend of serological tests and no major complication. Wright agglutination test, Coombs Wright, and 2ME all diminished to normal range of 1/40 after two years.

## Discussion

Brucellosis is often referred to as “ a disease with a thousand faces”, hence remaining as a major public health concern due to frequent misdiagnosis or underdiagnosis, economic burden, and serious complications [4]. A novel estimation model suggested the true incidence may be as high as 2.1 million new cases of brucellosis annually, with an estimated incidence of half a million cases globally [5]. This communicable disease particularly affects endemic regions like the Middle East, the Mediterranean regions, Central Asia, China, India, Africa, and parts of Central and South America [6]. It is one of the momentous health problems in Iran as well, where the incidence rates reach as high as 225 cases in 100,000 population in most endemic areas. Iran currently has the second-highest prevalence of brucellosis in the world [7]. Increasing age at disease onset and prolonged duration of illness have been associated with a higher risk of developing neurobrucellosis [8].

Transmission mainly occurs through unpasteurized dairy products or straight contact with infected animals [9], [10]. Inflammatory processes play a crucial role in the pathogenesis of human brucellosis. Inflammatory infiltrates composed of lymphocytes, monocytes, and neutrophils are commonly identified in affected organs [11], [12]. The central nervous system is supposedly affected through hematogenous dissemination via infected monocytes transporting the bacteria to different types of brain cells like astrocytes and microglia. Experimental studies demonstrated the subsequent astrogliosis leads to secretion of various cytokines and chemokines, particularly IL-6 upregulates microglial phagocytic capacity and contributes to neuronal damage and consequential neurological manifestations [11]. As a multisystemic infection, it may involve both the central and peripheral nervous systems [13].

Although not a frequent complication, neurobrucellosis is associated with substantial morbidity [13]. It often presents with miscellaneous neurological syndromes and is often misdiagnosed. Headache, fever, decreased consciousness, and seizures are frequent symptoms, which occur in more than half of patients. Other less common manifestations were listed as altered deep tendon reflexes muscular weakness, hemiparesis, nausea and vomiting, auditory impairment, papilledema, diplopia, and gait disturbances. Meningeal irritation signs were the most frequent on examination [14]. Myelopathy may occur due to Brucella spondylitis, which can cause vertebral erosion and collapse leading to cord compression [15]. Similar to us, Pourmontaseri et al. [16] described an intriguing neurobrucellosis case with paraplegia and incontinence in conjunction with other neurologic deficits. Although aphasia manifestation is rarely reported, there are documented cases suggesting that neurobrucellosis can involve dominant hemisphere language networks and produce aphasic syndromes. Moreover, global aphasia reflects severe impairment of the MCA territory of the dominant cerebral hemisphere, including Broca’s area (inferior frontal gyrus) and Wernicke’s area (posterior superior temporal gyrus). Such lesions result in profound deficits in both language comprehension and expression (characteristic of global aphasia) even in the absence of isolated focal lesions on standard imaging [17]. For instance, in a similar case series with suspected ischemic stroke and recurrent aphasia alongside progressive dysfunction of spinal motor pathways, neurobrucellosis was confirmed via CSF culture and advanced molecular diagnostics [18]. Overall, such findings along with even less common concurrence of our case highlight the multifocal nature of neurobrucellosis as it affects both cerebral and spinal structures and development of inflammatory small and medium-sized vasculitis that lead to ischemic injury [19].

Diagnosis cannot be established solely on clinical manifestations given the nonspecific and variable manifestations. Therefore, different laboratory modalities are implemented [20]. Although Brucella grows slowly, culture remains the gold standard as it provides definitive diagnosis in low clinical suspicion due to non-pathognomic manifestations. It also enables precise identification and antibiotic susceptibility testing, even in cases where serology is negative in the early stages [21]. Serological tests have limitations due to low sensitivity and none is fully reliable for definitive diagnosis, though they are widely applied [14], [22]. PCR is particularly valuable in complicated cases, including neurobrucellosis (in which the serological test often fails) and serves as an alternative confirmatory method to culture [23]. Radiologic findings in neurobrucellosis range from normal (most frequent) to evidence of white matter lesions, meningeal inflammation, and vascular or cranial nerve involvement. The vestibulocochlear nerve is the most commonly affected cranial nerve in neurobrucellosis. Enhancement of the perivascular space or lumbar nerve roots and granulomatous lesions are less common. White matter hyperintensities on T2-weighted MRI and features of demyelination have also been described [14], [24], [25].

Treatment is challenging due to relapsing nature of brucellosis. Monotherapy and short treatment courses are associated with higher risk of relapse. Treatment of first choice for uncomplicated brucellosis consists of dual therapy with doxycycline and streptomycin, which has shown the highest efficacy and lowest relapse rate. However, streptomycin does not show any significant advantage over gentamicin. Potential alternative regimens (rifampicin plus doxycycline or doxycycline plus sulfamethoxazole-trimethoprim regimens) carry higher therapeutic failure and relapse risks, hence they are considered as the second choice of treatment [26], [27], [28], [29]. Quinolones have also been considered. Triple therapy with doxycycline, rifampicin, and an aminoglycoside achieves even superior outcomes in comparison with dual regimens [27]. Recent evidence favors the use of combination of three or more agents in neurobrucellosis and antibiotic choice must ensure adequate central nervous system penetration. Doxycycline, sulfamethoxazole-trimethoprim, rifampicin, and ceftriaxone are considerable choices. Quinolones (ciprofloxacin) might also be considered in combination therapy, though relapse rates are higher [30], [31], [32], [33]. Treatment duration ranges from at least 6 weeks to 18 weeks, and in many cases up to 6 months in endemic regions such as Iran. Triple therapy with ceftriaxone (4 g/day), rifampicin at 15 mg/kg/day (600–900 mg), and doxycycline (200 mg/day) has been recommended to reduce relapse risk [34]. The treatment should be continued until CSF parameters normalize [35], [36].

As presented, the patient endured a prolonged course of the disease with no precise diagnosis, as many brucellosis manifestations overlap with other differential diagnosis. The patient’s main complaints of low back pain and progressive lower-limb weakness initially overlapped with neurological or rheumatologic disorders. The patient did not report any history of prominent fever (a typical hallmark of brucellosis), which further complicated and delayed the diagnosis of brucellosis. Wright agglutinin test for Brucella spp. became positive. MRIs revealed ischemic disease consistent with aphasia and spondylodiscitis at the T6-T7 and L1–L2 levels. Ultimately, positive serology and CSF culture for Brucella spp. established the diagnosis of neurobrucellosis. The patient was then treated with rifampicin, gentamicin, ceftriaxone, and doxycycline following confirmation of Brucella infection, in accordance with regimens shown to reduce relapse risk.

## Conclusion

Brucellosis is known as “a disease with a thousand faces”. It is classically described as a prolonged intermittent fever and may not be readily suspected when this hallmark symptom is absent, particularly when neurological complications like paraplegia and aphasia are prominent. In such cases, history of occupational exposure to contaminated sources, residence in or travel to endemic areas, persistent unexplained symptoms, and the lack of an alternative diagnosis should raise clinical suspicion. Since the multifocal presentation of neurobrucellosis mimics a wide spectrum of both cerebral and spinal disorders ranging from a Guillain-Barré-like syndrome to an acute cerebrovascular event due to vasculitis mechanisms, thorough investigation like serological tests and CSF analysis should be pursued in patients from endemic areas with unresolved neurological manifestations. Early diagnosis and prompt antibiotic regimen with adequate blood-brain barrier penetration are essential to prevent irreversible neurological sequelae, particularly in neurological involvement of elderly patients.

## CRediT authorship contribution statement

**Amir Ehtemami:** Writing – review & editing, Methodology. **Kamyar Khazaei:** Writing – original draft, Data curation, Conceptualization. **Negar Abbasi Jamaat:** Writing – original draft, Data curation. **Yasamin Pourabedini Aghdam:** Writing – original draft, Data curation. **Davood Sanaei Delir Zavaragh:** Supervision, Project administration.

## Consent

Written informed consent was obtained from the patient for anonymous publication and accompanying images. A copy of the written consent is available for review by the Editor-in-Chief on request.

## Ethical approval

Approval of the Ethical Standards Board of Farhikhtegan Hospital was obtained. The study adhered to the tenets of the Declaration of Helsinki and its later amendments.

## Declaration of Generative AI and AI-assisted technologies in the writing process

The authors used AI-based tools only for language editing and grammar checking. No content was generated by AI, and the authors take full responsibility for the integrity and originality of the manuscript.

## Funding

No public or commercial funding.

## Declaration of Competing Interest

The authors declare that they have no competing interests.

## Data Availability

The datasets generated and analyzed during the current study are available from the corresponding author on reasonable request.
